# Microarray analysis of E9.5 reduced folate carrier (*RFC1; Slc19a1*) knockout embryos reveals altered expression of genes in the cubilin-megalin multiligand endocytic receptor complex

**DOI:** 10.1186/1471-2164-9-156

**Published:** 2008-04-09

**Authors:** Janee Gelineau-van Waes, Joyce R Maddox, Lynette M Smith, Michael van Waes, Justin Wilberding, James D Eudy, Linda K Bauer, Richard H Finnell

**Affiliations:** 1 Department of Genetics, Cell Biology & Anatomy, University of Nebraska Medical Center, Omaha, NE 68198-5455, USA; 2 Department of Preventive and Societal Medicine, University of Nebraska Medical Center, Omaha, NE 68198-4350, USA; 3 LI-COR Biosciences Inc., Lincoln, NE 68504, USA; 4 Center for Environmental and Genetic Medicine, Institute of Biosciences and Technology, Texas A&M University System Health Science Center, Houston, Texas 77030, USA

## Abstract

**Background:**

The reduced folate carrier (*RFC1*) is an integral membrane protein and facilitative anion exchanger that mediates delivery of 5-methyltetrahydrofolate into mammalian cells. Adequate maternal-fetal transport of folate is necessary for normal embryogenesis. Targeted inactivation of the murine *RFC1 *gene results in post-implantation embryolethality, but daily folic acid supplementation of pregnant dams prolongs survival of homozygous embryos until mid-gestation. At E10.5 *RFC1*^-/- ^embryos are developmentally delayed relative to wildtype littermates, have multiple malformations, including neural tube defects, and die due to failure of chorioallantoic fusion. The mesoderm is sparse and disorganized, and there is a marked absence of erythrocytes in yolk sac blood islands. The identification of alterations in gene expression and signaling pathways involved in the observed dysmorphology following inactivation of RFC1-mediated folate transport are the focus of this investigation.

**Results:**

Affymetrix microarray analysis of the relative gene expression profiles in whole E9.5 *RFC1*^-/- ^vs. *RFC1*^+/+ ^embryos identified 200 known genes that were differentially expressed. Major ontology groups included transcription factors (13.04%), and genes involved in transport functions (ion, lipid, carbohydrate) (11.37%). Genes that code for receptors, ligands and interacting proteins in the cubilin-megalin multiligand endocytic receptor complex accounted for 9.36% of the total, followed closely by several genes involved in hematopoiesis (8.03%). The most highly significant gene network identified by Ingenuity™ Pathway analysis included 12 genes in the cubilin-megalin multiligand endocytic receptor complex. Altered expression of these genes was validated by quantitative RT-PCR, and immunohistochemical analysis demonstrated that megalin protein expression disappeared from the visceral yolk sac of *RFC1*^-/- ^embryos, while cubilin protein was widely misexpressed.

**Conclusion:**

Inactivation of *RFC1 *impacts the expression of several ligands and interacting proteins in the cubilin-amnionless-megalin complex that are involved in the maternal-fetal transport of folate and other nutrients, lipids and morphogens such as sonic hedgehog (Shh) and retinoids that play critical roles in normal embryogenesis.

## Background

Folate and vitamin B12 are essential vitamins derived from various food sources that play an important role in erythropoiesis, DNA biosynthesis, and embryogenesis. Nutritional deficiencies or genetic variations that impact folate and/or vitamin B12 homeostasis may result in megaloblastic anemia [1], and failure of maternal-fetal transport of these nutrients has been shown to adversely impact normal embryogenesis [2-5]. Folate is a vitamin derived from plant sources that consists of a pteridine ring structure attached to a para-aminobenzoic acid side chain. Tetrahydrofolate, the reduced form of folate, functions as an important co-factor for donating or accepting methyl groups. Adequate levels of folate for the incorporation of single carbon groups into purine, or methylation of deoxyuridylate to form thymidylate, are necessary for DNA biosynthesis. Vitamin B12, also known as cobalamin, is another important vitamin that is obtained from dietary sources such as meat, fish and soybean. Folate and cobalamin metabolism are highly interdependent. In mammals, cobalamin functions as a co-enzyme for methionine synthase, which is involved in the tetrahydrofolate-dependent methylation of homocysteine to methionine [6]. According to the "folate-trap hypothesis", cobalamin deficiency results in 'trapping' of tetrahydrofolate, producing a functional folate deficiency that leads to impaired erythropoiesis and DNA biosynthesis [7].

The primary mechanisms for folate delivery into the cell are through (1) carrier-mediated (reduced folate carrier; RFC1) or (2) receptor-mediated (folate receptor; Folr1) processes. These two transport systems are distinguished by their unique patterns of tissue expression, differing protein structures, specificities for oxidized vs. reduced folates, and divergent mechanisms for transmembrane transport of folates [8]. As mammalian cells are not capable of synthesizing folates *de novo*, these two transport systems play a critical role in mediating folate uptake for the biosynthesis of purines, pyrimidines, and certain amino acids that are necessary for cell survival and proliferation. It has recently been discovered that megalin, a large multiligand endocytic receptor of the low density lipoprotein receptor family, is capable of binding and mediating uptake of the soluble form of the GPI-anchored folate receptor Folr1, providing yet another pathway for the cellular internalization of folate [9].

The principal route of folate transport into mammalian cells is via the reduced folate carrier (RFC1), a bidirectional transporter characterized by twelve transmembrane domains [8,10]. RFC1 preferentially transports reduced folates, such as N^5^-methyltetrahydrofolate or N^5^-formyltetrahydrofolate, which are anionic at physiological pH. It functions as a facilitative anion exchanger, capable of transporting anionic folates into the cell via a co-transport system in which the uphill transport of folate is coupled to the downhill efflux of organic phosphates or sulfates [8,11]. Folate uptake into intestinal cells is markedly increased at pH 5.5 relative to pH 7.4 [12,13], suggesting that RFC1-mediated folate uptake is highly dependent on acidic extracellular pH. In adult tissues, RFC1 expression is observed in the brush border of the small and large intestine, and in the basolateral membrane of renal tubular epithelium, as well as in hepatocytes, choroid plexus, and the retinal pigment epithelium [14]. During development, RFC1 is highly expressed in multiple tissue types, including the placenta and yolk sac, neural tube, craniofacial region, limb buds and heart [14,15].

We have recently characterized the role of *RFC1 *during embryonic development in a mouse model in which *RFC1 *was inactivated by homologous recombination [16]. Without maternal folate supplementation, *RFC1*^-/- ^embryos die *in utero *shortly after implantation. *RFC1*^-/- ^embryos harvested from dams receiving low doses of supplemental folic acid (25 mg/kg/day SQ), survive to mid-gestation (E10.5), but are developmentally delayed, and display multiple malformations, including severe neural tube defects, craniofacial, heart, and limb abnormalities. Examination of the placenta reveals that embryolethality is due to a failure of chorioallantoic fusion. The fetal vasculature appears to form normally, but there is a pronounced absence of erythropoiesis, with very few nucleated fetal red blood cells present in either the fetal blood vessels or the yolk sac blood islands. Maternal folate supplementation with 50 mg/kg/day results in survival to term of 22% of the *RFC1 *mutants, although the surviving fetuses present with multiple malformations of the craniofacies, lungs, heart and skin.

The reduced folate carrier gene has previously been inactivated in a mouse model [17] through targeted disruption of a large part of exon 3. Zhao [17] reported that the *RFC1*^-/- ^embryos died *in utero *before E9.5, but near-normal development could be sustained by supplementing pregnant *RFC*^+/- ^dams with daily doses of 1 mg folic acid SQ. Approximately 10% of the partially rescued *RFC1*^-/- ^pups were live-born, but all died within 12 days post-partum. In the Zhao [17]*RFC1 *knockout mouse model, perinatal lethality was attributed to a marked absence of erythropoiesis in bone marrow, spleen, and liver. Our recent findings indicate that inactivation of *RFC1 *results in congenital malformations of the heart and lungs that undoubtedly contribute to the failure of *RFC1 *mutants to survive postnatally.

The purpose of this report is to identify alterations in biochemical and/or molecular genetic pathways that may play a role in the abnormal embryonic morphogenesis observed following inactivation of RFC1. In the current study, the gene expression profiles of whole E9.5 *RFC1*^-/- ^embryos were compared to that of *RFC1*^+/+ ^littermates harvested from pregnant dams receiving daily low dose (25 mg/kg/day S.Q.) folate supplementation. Our microarray analysis shows that in the absence of functional *RFC1*, the expression profiles of numerous transcription factors, as well as several genes involved in hematopoiesis, ion homeostasis, and genes encoding for receptors and/or ligands in the cubilin-megalin multiligand endocytic receptor complex are significantly altered. Follow-up quantitative RT-PCR validation confirms alterations in the expression profiles of several ligands and interacting proteins in the cubilin-megalin complex that are involved in nutrient transport of vitamins and lipids to the developing embryo.

## Results

### Microarray analysis

In the microarray analysis of E9.5 *RFC1*^-/- ^embryos vs. *RFC1*^+/+ ^embryos, the expression patterns of 783 genes were found to be significantly altered at the 0.001 level. However, when looking at the false discovery rate (FDR), only 288 genes (probe sets) were significantly altered at the 0.001 level. Several of the probe sets represent alternate transcripts for the same gene. Taking this into account, 250 unique genes were identified as differentially expressed in the microarray analysis. Of these, 200 represent known genes, while 50 are ESTs or unknowns. The 12 known genes in our data set that have been identified as cell surface receptors, ligands, or interacting proteins in the cubilin-megalin multiligand endocytic receptor complex are listed in Table 1, and the complete set of 200 known genes that were differentially expressed following statistical analysis of the microarray data are also provided [see Additional file 1]. The genes have been categorized according to ontology groups, and the file contains the accession number, gene symbol, gene name, and the mean fold change for the expression level of each gene. The fold-change represents the value for the comparison of level of gene expression in the *RFC1*^-/- ^embryos relative to the *RFC1*^+/+ ^embryos. (The data discussed in this publication have also been deposited in NCBIs Gene Expression Omnibus [18] and are accessible to the public through GEO Series accession number GSE10659).

**Table 1:** Microarray gene list: Multiligand endocytic receptor complex

***RFC1, E 9.5, whole embryo, nullizygote vs wildtype, Affymetrix mouse 430_2 microarray results***			
***RefSeq Transcript ID***	***Gene Symbol Affy***	***Description***	***Fold Change***

NM_009692	Apoa1	apolipoprotein A-I	65.97
XM_130038	Cubn	cubilin (intrinsic factor-cobalamin receptor)	35.49
NM_018816	Apom	apolipoprotein M	23.41
NM_013697	Ttr	transthyretin	18.02
NM_011255	Rbp4	retinol binding protein 4, plasma	14.43
NM_134249	Timd2	T-cell immunoglobulin and mucin domain containing 2	12.04
NM_133977	Trf	transferrin	9.41
NM_009696	Apoe	apolipoprotein E	4.70
NM_001008702///NM_023118	Dab2	disabled homolog 2 (Drosophila)	3.78
NM_008662	Myo6	myosin VI	3.56
NM_017399	Fabp1	fatty acid binding protein 1, liver	3.10
NM_013587	Lrpap1	low density lipoprotein receptor-related protein associated protein 1	2.16

### Distribution of gene ontology groups

**Figure 1:**
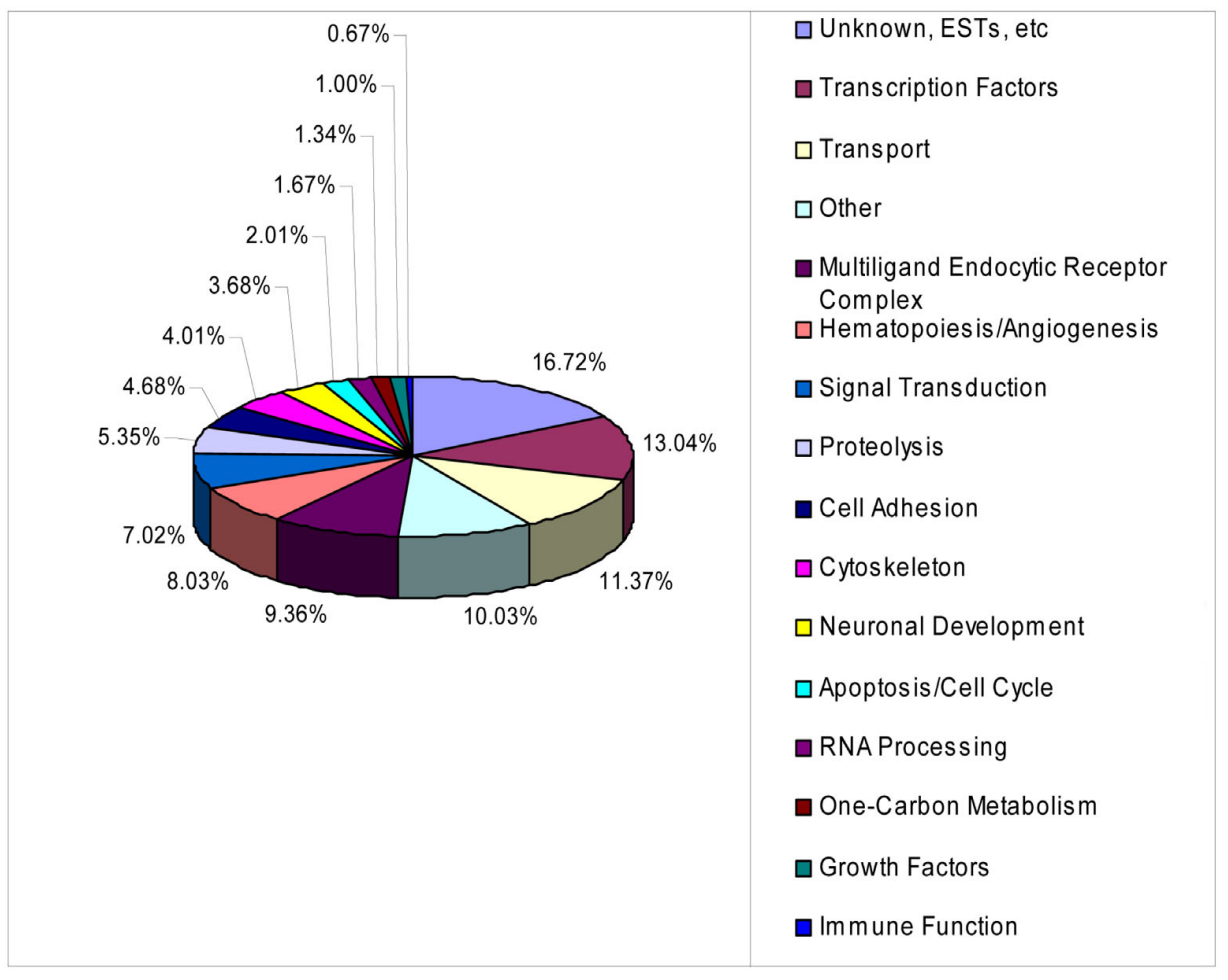
**Distribution of gene ontology groups**. A pie chart showing the relative distribution (percentage) of the gene ontology groups identified in the microarray analysis of E9.5 *RFC1*^-/- ^vs. *RFC1*^+/+ ^embryos is shown. A complete list of the genes within each of these gene ontology groups that were differentially expressed following statistical analysis of the microarray data is provided in [Additional file 1].

A pie chart showing the relative distribution (percentage) of the gene ontology groups identified in the microarray analysis of the *RFC1*^-/- ^vs. *RFC1*^+/+ ^embryos is shown in Figure 1. The largest ontology group of known genes that was altered included several families of transcription factors (13.04%), followed by genes involved in transport functions (ion, lipid, carbohydrate) (11.37%). Genes that code for receptors, ligands and interacting proteins in the cubilin-megalin multiligand endocytic receptor complex account for 9.36% of the total, followed closely by several genes involved in hematopoiesis (8.03%).

### Ingenuity Pathway Analysis

**Figure 2:**
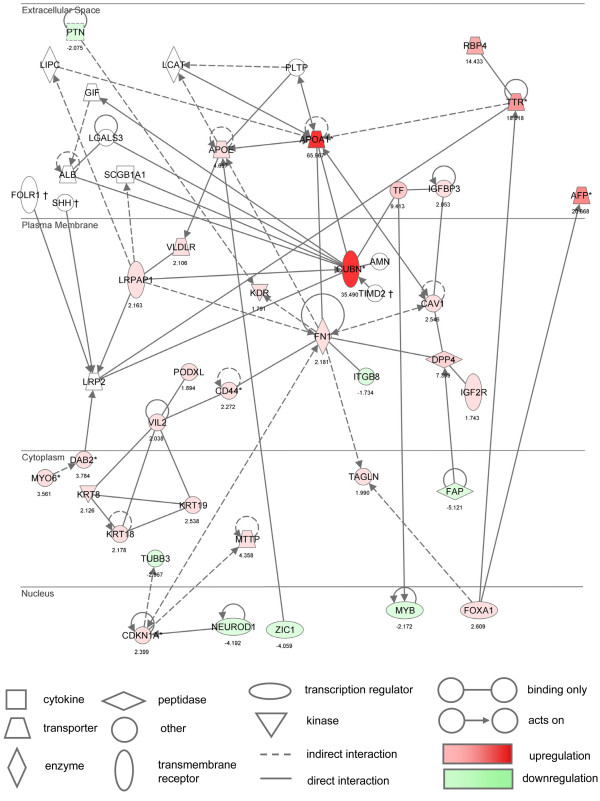
**Ingenuity™ Pathway gene network**. The most highly significant gene network identified in the Ingenuity™ Pathway analysis of our Affymetrix microarray data is shown. An additional 11 'custom' genes were placed into this network based on new information available in the recent literature. Many of the key genes highlighted in this top network are members of the ontology group that includes receptors, ligands and interacting proteins found in the cubilin-megalin multiligand endocytic receptor complex. The networks are displayed graphically as nodes (genes/gene products) and edges (the biological relationships between the nodes). The intensity of the node color indicates the degree of up- (red) or down- (green) regulation in gene expression in our microarray analysis. Nodes are displayed using various shapes that represent the functional class of the gene product. Edges are displayed as a direct interaction (solid line) or as an indirect/unknown interaction (dashed line). The nodes have also been arranged to show the location of the gene/gene product with respect to cellular or subcellular localization (e.g. plasma membrane, cytoplasm, nucleus, etc.).

The most highly significant gene network identified in the Ingenuity Pathway analysis (Ingenuity™ Systems, Inc., Mountain View, CA) of our Affymetrix microarray data is shown in Figure 2. Based on new information available in the recent literature (interactions not yet reported in the latest release of the Ingenuity Knowledge Base), we identified an additional 11 genes of interest that were placed into this network. These additional genes are represented by 11 custom nodes and edges. In the original "Network 1" of the Ingenuity Pathway Analysis, 32 of the 34 genes that appear were statistically up or down-regulated on our microarray analysis, giving it a score of 55. With the addition of the custom nodes/edges (*Folr1, Shh, Timd2, Myo6, Dab2*) and nodes from the Cubn neighborhood–(Tf*, LgalS3, Alb, Gif, Amn, Lrp2, Lrpap1*, ScgB1A1) {*genes that already appear in the default "Network 1"} 34 of the 46 genes in the custom network were found to be significant. Many of the key genes highlighted in this top network are members of the ontology group that includes receptors, ligands and interacting proteins found in the cubilin-megalin multiligand endocytic receptor complex. This complex became the focus of further studies due to its important role in the uptake of folate and other vitamins, lipids, and nutrients essential for embryonic development.

### Cubilin-megalin multiligand endocytic receptor complex

**Figure 3:**
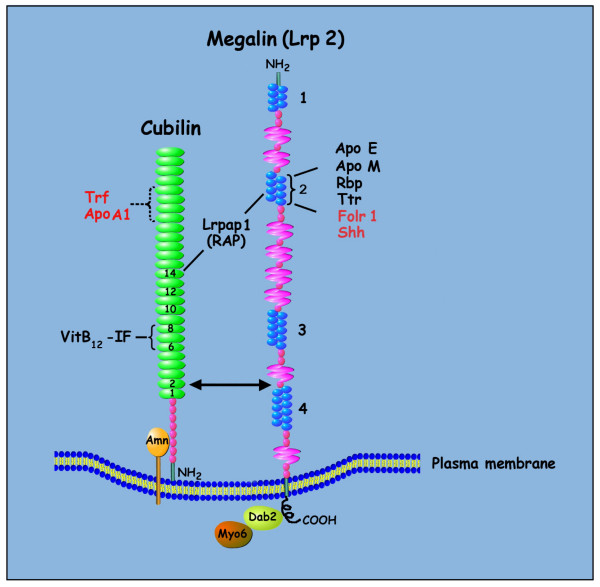
**Cubilin-megalin multiligand endocytic receptor complex**. A cartoon diagram of the structure of the cubilin-amnionless-megalin multiligand receptor endocytic complex [19] is shown. Megalin is a cell-surface receptor/transporter consisting of a large extracellular region, a single transmembrane domain, and a C-terminal cytoplasmic tail. The extracellular domain of megalin contains four clusters of lipoprotein receptor ligand-binding repeats (blue), growth factor repeats, an EGF repeat, and YWTD spacer regions. The second cluster of ligand-binding repeats has been identified as a common binding site for several ligands including apolipoprotein E (Apo E), apolipoprotein M (Apo M), retinol binding protein (Rbp), and transthyretin (Ttr). Megalin also binds the soluble form of the folate receptor (Folr1), and the morphogen sonic hedgehog (Shh). The cytoplasmic tail of megalin binds Dab2, a cytosolic adapter protein important for megalin-mediated endocytosis, and Dab2 binds and recruits Myo6 to clathrin-coated vesicles. The receptor-associated protein (Lrpap1; RAP) binds both megalin and cubilin. Cubilin is a peripheral membrane receptor comprised of a short amino terminal, eight EGF type domains, and 27 CUB domains (green). The amino-terminal end of cubilin is attached to the extracellular part of amnionless (Amn), and amnionless provides the transmembrane domain necessary for the anchoring and endocytic trafficking of cubilin. Cubilin ligands include transferring (Trf), albumin, hemoglobin, apolipoprotein A1 (ApoA1), and intrinsic factor (IF)-vitamin B_12_.

A diagram of the structure of the cubilin-amnionless-megalin multiligand receptor endocytic complex is shown in Figure 3[19]. Megalin and cubilin are two multifunctional endocytic receptors that act in concert in certain tissues to mediate the uptake of a variety of lipoproteins and vitamin-carrier complexes [19-21]. Striking similarities in temporal and spatial expression patterns of megalin and cubilin have been described in mid-late gestation rat embryos [22], although differential patterns of expression have also been reported during early embryonic development in the mouse [23]. Megalin (Lrp2) is a low-density lipoprotein receptor-related protein that is co-expressed with cubilin in many absorptive epithelia, including the neuroepithelium and yolk sac of developing embryos. Several studies indicate a critical role for megalin in normal embryogenesis [24]. Megalin has been shown to bind and internalize multiple ligands, including the soluble folate binding protein (Folr1), and sonic hedgehog (Shh), among others [19,25-29]. Lack of megalin receptor function in a knockout mouse model results in abnormalities of the kidneys, lungs, central nervous system and craniofacies, and megalin-deficient pups die perinatally within the first few minutes of life due to respiratory insufficiency [30]. Conditional inactivation of megalin in the neuroepithelium has been shown to result in a loss of Shh expression in the ventral telencephalon [31], indicating that megalin-mediated uptake of Shh in the neuroepithelium is critical for early forebrain development [32].

Cubilin (gp280) functions as an endocytic receptor for the intrinsic factor-cobalamin complex in the intestine, and as a receptor for apolipoprotein A1 and albumin reabsorption in the kidney proximal tubules and visceral yolk sac [33]. Cubilin is a peripheral membrane receptor comprised of a short amino terminal amphipathic helix, eight EGF type domains, and 27 amino acid modules known as CUB domains [34]. Although cubilin lacks a classic transmembrane domain and cytoplasmic tail, the amino-terminal end is necessary for membrane anchoring [35]. The amino-terminal region of cubilin is attached to the extracellular part of *amnionless*, which provides the transmembrane domain necessary for anchoring and endocytic trafficking of cubilin [36]. Antibodies directed against cubilin have been reported to have teratogenic effects in rats [37], and targeted disruption of cubilin in a mouse model reveals an essential role for this receptor in embryogenesis [38]. Homozygous inactivation of the murine cubilin gene results in embryolethality between E7.5 – E13.5. Cubilin knockout mice are characterized by defects in certain mesodermally-derived tissues, including failure of somite formation and abnormalities of the yolk sac blood islands and visceral endoderm [38].

A comparative overview of the developmental phenotype of mice with genetically engineered deletions in critical components of the cubilin-megalin multiligand endocytic receptor complex including *Cubilin, Amnionless, Megalin, Receptor-Associated Protein (RAP; Lrpap1)*, and *Disabled homolog-2 *is provided [see Additional file 2]. The phenotypes of these mutants are compared to the developmental anomalies observed following genetically engineered deletions in the folate transporters *Folr1 *and *RFC1*. *RFC1*^-/- ^embryos on low dose maternal folate supplementation (such as those examined in the microarray analysis) display certain phenotypic traits in common with the more severely affected *cubilin, amnionless*, and *Dab2 *knockouts, including failure of chorioallantoic fusion and defects in mesoderm-derived structures, whereas *RFC1*^-/- ^embryos that survive to term following high dose maternal folate supplementation display a range of malformations more similar to the *megalin *knockout mutants.

### Quantitative real-time PCR validation

**Figure 4:**
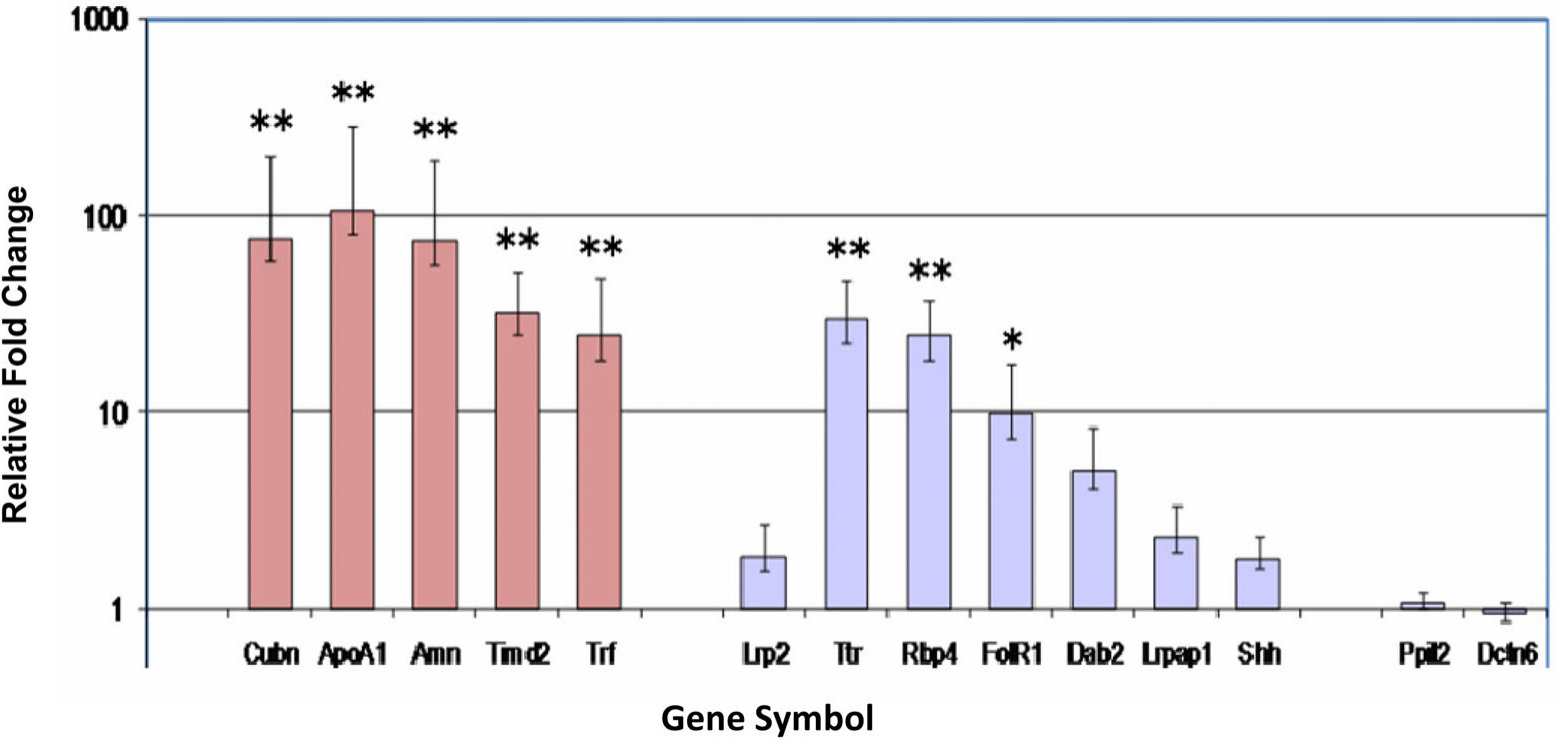
**Quantitative RT-PCR relative gene expression levels**. The results of the quantitative RT-PCR validation experiments are shown in the graph. Genes were selected for the RT-PCR analysis based on the results of the microarray analysis, or because of their presence in the cubilin-megalin multiligand endocytic receptor complex. The red bars on the left represent the fold change in the expression of cubilin and cubilin-related genes and the blue bars on the right represent the fold change in the expression of megalin and megalin-related genes relative to the expression of the housekeeping genes *Ppil2 *and *Dctn6*. (*p < .05; **p < .01) Error bars represent SEM values. Gene symbols, relative expression levels and p values are provided in the corresponding Table 2. *Cubilin (Cubn), Apolipoprotein A1 (ApoA1), Amnionless (Amn), Transferrin (Trf), T-cell immunoglobin and mucin domain containing 2 (Timd2), Megalin (Lrp2), Lipoprotein receptor-associated protein 1 (Lrpap1), Disabled (Dab2), Folate Receptor (Folr1), Retinol Binding Protein 4 (Rbp4), Sonic Hedgehog (Shh), Transthyretin (Ttr)*.

Genes in the cubilin-megalin multiligand endocytic receptor complex identified in the Affymetrix microarray analysis, Ingenuity Pathway analysis, or through additional information available in the literature were selected for further expression analysis by quantitative RT-PCR (qRT-PCR). The twelve genes selected for qRT-PCR validation of alterations in gene expression are listed along with a description of their function, and the ABI primer/probe sets used for these experiments [see Additional file 3]. Figure 4 and Table 2 list the results of the qRT-PCR analysis, including p-values, and a graph of the relative levels of expression of the selected genes in the multiligand receptor endocytic complex is shown. *Cubn, ApoA1, Amn, Trf, Timd2, Rbp4, Ttr*, and the GPI-anchored folate receptor *(Folr1) *are significantly upregulated in E9.5 *RFC1*^-/- ^embryos relative to E9.5 *RFC1*^+/+ ^littermates (*p < .05; **p < .01). However, the qRT-PCR analysis did not detect statistically significant changes in the expression of *Lrp2, Lrpap1, Dab2*, or *Shh*. Error bars represent SEM values.

**Table 2:** qRT-PCR Gene Expression Levels

**Gene**	**Relative Expression**	**P value**
Cubn	76.6	0.002
ApoA1	106.4	0.001
Amn	75.3	0.002
Trf	24.6	0.005
Timd2	32.7	0.003
Lrp2	1.9	0.462
Lrpap1	2.3	0.279
Dab2	5.1	0.051
Folr1	10.0	0.017
Rbp4	24.8	0.004
Shh	1.8	0.496
Ttr	29.8	0.004
Ppil2	1.1	1.000
Dctn6	0.9	0.998

Although neither *Folr1 *nor *megalin *gene expression made the cut-off for statistical significance on our microarray analysis, we included them in our qRT-PCR analysis because of their presence in the multiligand endocytic receptor complex, and their important role in nutrient delivery to the developing embryo (*Folr1 *was upregulated 5.3 fold, p < .0003 on the microarray analysis, but did not make the cut-off for the FDR level). qRT-PCR results demonstrated no significant difference in message levels of megalin between the two groups, but a 10-fold increase in *Folr1 *expression was detected (p < .017) in *RFC1*^-/-^relative to *RFC*^+/+ ^littermates. RT-PCR validation also demonstrates a highly significant upregulation of *cubilin*message in *RFC1*^-/- ^embryos (77-fold, p < .002). *Amn *mediates cell-surface localization and endocytic function of cubilin in the visceral endoderm [39]. *Amn *did not make the list of 288 genes identified as statistically significant in our microarray analysis using the stringent cut-off of 0.001 for adjusted p-values (p = .00109). However, due to its critical role in cubilin expression and function, we chose to further analyze *amnionless *gene expression levels by quantitative RT-PCR. Our results show that, similar to cubilin, the expression of *amnionless *was highly significantly upregulated (75-fold; p < .002) in *RFC1*^-/- ^embryos.

### Immunohistochemistry

During normal embryonic development, megalin and cubilin are highly expressed in the visceral endoderm (VE) and visceral yolk sac (VYS), and function cooperatively in the maternal-fetal transfer of multiple nutrients and morphogens. The VE is a polarized epithelial tissue that surrounds the developing epiblast. The apical surface of the VE faces maternal tissues, and contains numerous microvilli that function to absorb nutrients from the maternal environment [40]. Additional roles in patterning anterior-posterior polarity of the early embryo, correct positioning of the primitive streak, induction of blood precursors, and forebrain development have been ascribed to the anterior VE [41-43]. Following implantation, the VE surrounding the epiblast differentiates to form the VYS. The VYS continues to play a critical role in maternal-fetal nutrient and waste exchange prior to establishment of the chorioallantoic placenta [44].

**Figure 5:**
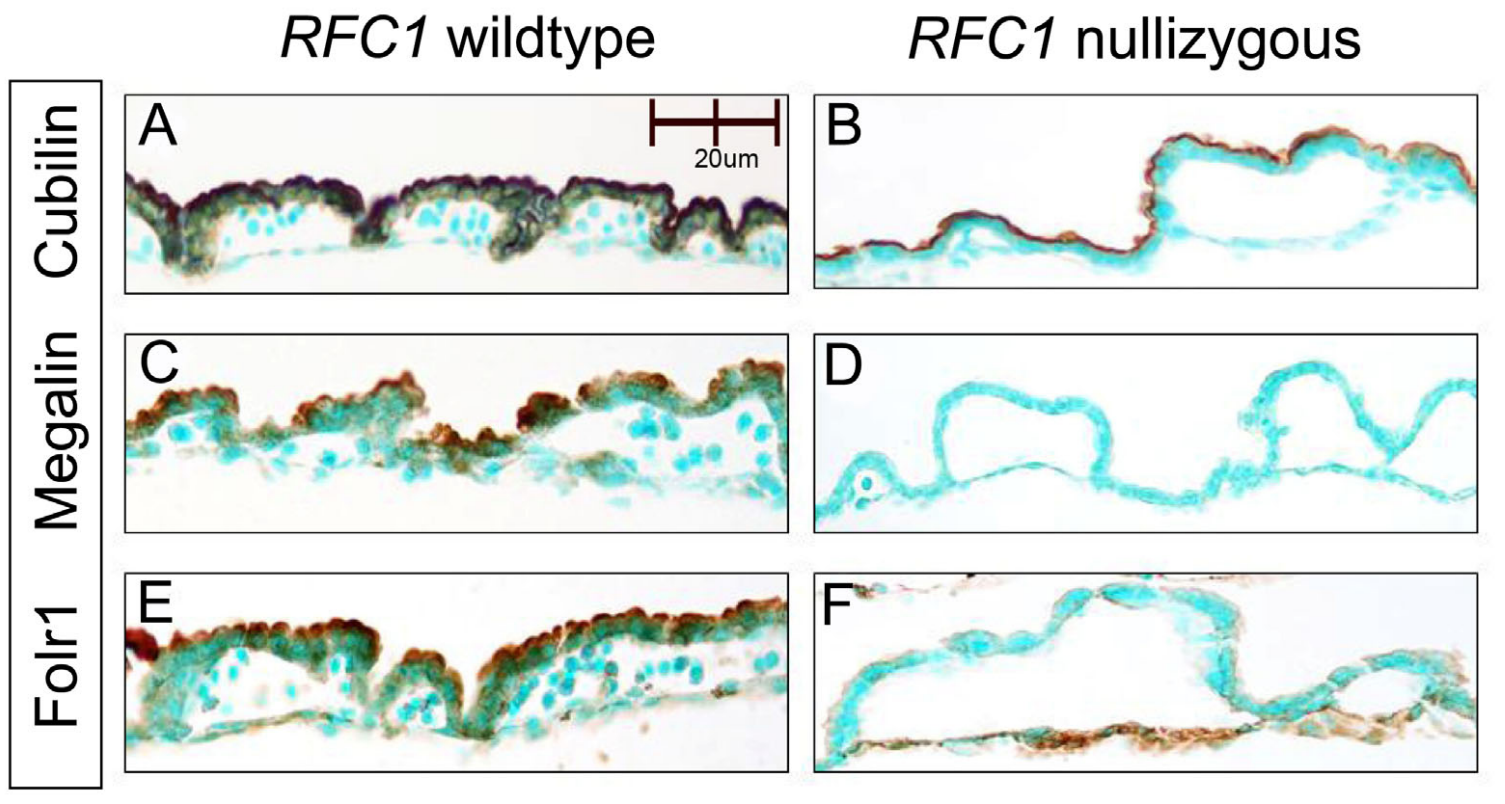
**Cubilin, megalin and Folr1 protein expression in VYS**. Expression of cubilin, megalin and Folr1 protein in the visceral yolk sac is shown in Figure 5. Cubilin (**A**), megalin (**C**) and Folr1 (**E**) protein are all highly expressed on the apical side of the visceral yolk sac in E9.5 *RFC1*^+/+ ^embryos. Cubilin is also highly expressed on the apical side of the visceral yolk sac in E9.5 *RFC1*^-/- ^embryos (**B**). Megalin expression, however, is completely absent from the visceral yolk sac of *RFC1 *mutants (**D**). Folr1 protein expression is absent from the apical plasma membrane of the VYS, but demonstrates increased expression in the endothelial cell layer of the yolk sac blood islands (**F**).

Immunohistochemical expression of cubilin, megalin and Folr1 protein in E9.5 *RFC1*^+/+ ^and *RFC1*^-/- ^embryos and extra-embryonic membranes was examined. Expression of cubilin, megalin and Folr1 protein in the visceral yolk sac (VYS) is shown in Figure 5. Cubilin (**5A**), megalin (**5C**) and Folr1 (**5E**) protein are all highly expressed on the apical side of the VYS in E9.5 *RFC1*^+/+ ^embryos (brown staining). Cubilin is also highly expressed on the apical side of the VYS in E9.5 *RFC1*^-/- ^embryos (**5B**). Megalin expression, however, is completely absent from the VYS of *RFC1 *mutants (**5D**). Folr1 protein expression is absent from the apical plasma membrane of the VYS in *RFC1*^-/- ^embryos, but demonstrates increased expression in the endothelial cell layer of the yolk sac blood islands (**5F**).

**Figure 6:**
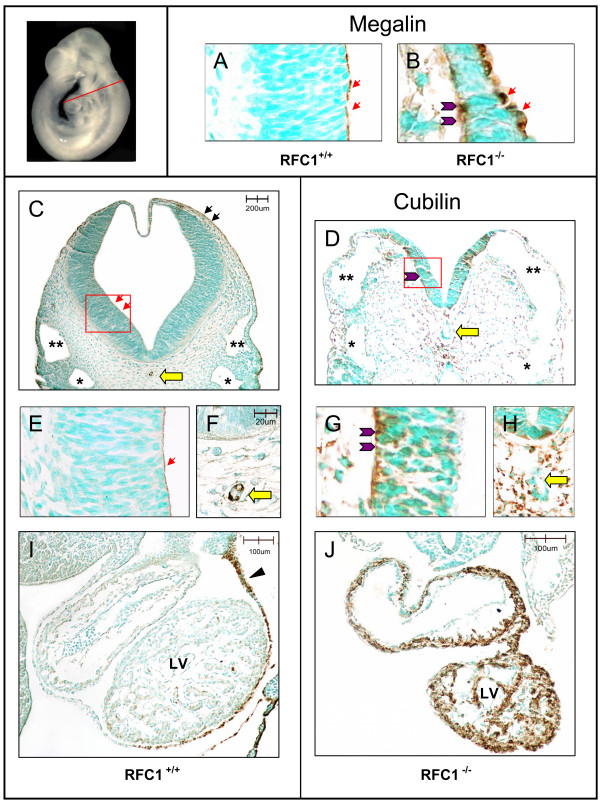
**Cubilin and megalin protein expression in E9.5 embryos**. Immunohistochemical staining was performed on transverse sections through E9.5 *RFC1*^+/+ ^and *RFC1*^-/- ^embryos in order to examine the relative expression and distribution of megalin and cubilin protein. The level and plane of the sections shown in Figure 6 is illustrated by the line drawn through the embryo shown in the upper left hand corner. The red boxes drawn over the neuroepithelium of embryos shown in **6C **and **6D **(4× magnification) indicate the orientation of the higher magnification panels (40× magnification) shown in **6A**, **6B**, **6E**, and **6G**; the apical surface (ventricular side) of the neuroepithelial cells is shown on the right of these panels, and the basolateral, or mesenchymal side of the neuroepithelial cells is shown on the left side of the panels. Megalin is expressed exclusively on the apical side of neuroepithelial cells in *RFC1 *wildtype embryos (**6A**; red arrows), but is expressed on both the apical (**6B**; red arrows) and the basolateral side (**6B**; purple arrowheads) of the neuroepithelium in *RFC1 *mutants. Cubilin expression is also restricted to the apical side of neuroepithelial cells in *RFC1 *wildtype embryos (**6C**, **6E**; red arrows), but is misexpressed on the basolateral side of the neuroepithelium in *RFC1 *nullizygous embryos (**6D**, **6G**; purple arrowheads). Staining for cubilin protein is visible in the surface ectoderm of *RFC1*^+/+ ^embryos (**6C**; black arrows), and discrete staining is also observed in the notochord (**6C**, **6F**; yellow arrow). Cubilin expression is highly upregulated in the cranial mesenchyme of *RFC1*^-/- ^embryos (shown in panel **6D**) relative to wildtype littermates, especially in the region surrounding the notochord (**6H**; yellow arrow). Staining for cubilin protein is visible in the mesothelial cells of the pericardium of *RFC1 *wildtype (**6I**; black arrowhead) and *RFC1 *mutant embryos (not visible in section shown in **6J**), and although there are a few cells that stain positive for cubilin in the trabeculae of the common ventricular chamber in the wildtype embryo, cubilin expression is dramatically upregulated throughout the myocardium of the *RFC1 *mutant (**6J**). Normal morphology of the primary head vein/cephalic extension of the anterior cardinal vein (**) and the third branchial arch artery (*) in the *RFC1 *wildtype embryo are shown in (6C); comparable structures labeled in the *RFC1 *mutant (6D) indicate abnormal vascular morphology. **LV: **left ventricle.

Expression of cubilin and megalin protein in the neuroepithelium is shown in Figure 6. Megalin expression is observed in the retinal pigment epithelium, cells lining the otic vesicle, and exclusively on the apical side of neuroepithelial cells (**6A**; red arrows) in E9.5 *RFC1*^+/+ ^embryos (brown staining). In *RFC1*^-/- ^embryos, however, megalin is expressed on both the apical (**6B**; red arrows) and basolateral side (**6B**; purple arrowheads) of the neuroepithelium. Positive staining for megalin is also observed in the surface ectoderm, notochord, pericardium, and endothelial cells lining vascular structures (primary head veins/cardinal veins and vitelline vein) of *RFC1*^+/+ ^embryos, but megalin staining is faint or absent in the (hindbrain) notochord and endothelium of similar vascular structures in *RFC1 *mutant embryos (not shown). Similar to megalin, cubilin expression is observed in the surface ectoderm (**6C**; black arrows), retinal pigment epithelium, otic vesicle, and apical side of neuroepithelial cells (**6C**; **6E**; red arrows) in E9.5 *RFC1*^+/+^embryos. In *RFC1*^-/- ^embryos, however, cubilin is misexpressed on the basolateral side of neuroepithelial cells (**6D; 6G; **purple arrowheads), Discrete, positive staining for cubilin is observed in the notochord of *RFC1*^+/+ ^embryos (**6F**, yellow arrow). In contrast, the expression of cubilin protein appears to be highly upregulated and widespread throughout the (sparse) mesenchyme underlying the neural tube (NT) of *RFC1*^-/- ^embryos, especially in the region *surrounding *the notochord (**6D, 6H**, yellow arrow). Cubilin staining is present in the pericardium of both *RFC1 *wildtype (**6I**; black arrowhead) and mutant embryos. Although there are a few cells that stain positive for cubilin in the trabeculae of the common ventricular chamber in the *RFC1 *wildtype heart, cubilin expression is dramatically upregulated throughout the myocardium of the developing heart in *RFC1 *mutants (**6J**).

## Discussion

In the present study, microarray analysis of gene expression in whole E9.5 *RFC1*^-/- ^embryos vs. *RFC1*^+/+ ^littermates was utilized as a tool for discovering genetic pathways disrupted in the absence of *RFC1 *that may play a role in the observed developmental anomalies. Our results indicate that *RFC1*^-/- ^embryos have alterations in the expression of several genes that code for receptors, ligands, interacting proteins, and modifiers of the cubilin-megalin multiligand endocytic receptor complex. Cubilin and megalin are both expressed in the VYS and play a critical role in the maternal-fetal transport of multiple ligands. In the VYS, blood vessels develop adjacent to the VE cell layer, and cell-cell interactions between yolk sac mesenchyme and VE appear to be important for vascular development [45,46]. Cubilin and megalin are cell surface proteins expressed in the extraembryonic VE that work cooperatively to mediate endocytosis of lipoproteins and vitamin complexes (including folate and intrinsic factor-vitamin B12) necessary for erythropoiesis and embryonic development. Uptake of folate and nutrients by the VE may therefore play a role in development of the adjacent yolk sac mesenchyme necessary for formation of the allantois and the initiation of erythropoiesis. In the absence of *RFC1*, the primary route of folate uptake into cells may be the GPI-anchored folate receptor, or internalization of folate bound to the soluble form of the folate receptor via endocytosis by megalin [28].

Although our RT-PCR results demonstrated no significant difference in message levels of megalin, a 10-fold increase in *Folr1 *expression was detected in *RFC1*^-/- ^relative to *RFC*^+/+ ^littermates. IHC examination of megalin and Folr1 protein expression in *RFC1*^-/- ^embryos indicates the disappearance of megalin on the apical cell surface of the VYS, as well as aberrant expression of Folr1, suggesting that, in addition to the absence of RFC1-mediated folate transport, folate uptake via the Folr1-megalin transport pathway may also be compromised in the VYS of *RFC1*^-/- ^embryos.

Microarray analysis and RT-PCR validation demonstrated highly significant upregulation of *cubilin*message in E9.5 *RFC1*^-/- ^embryos. Follow-up IHC analysis suggested a corresponding upregulation in cubilin protein, although technical challenges associated with extracting sufficient amounts of protein from the tiny E9.5 mutant embryos precluded western blot quantification. In *RFC1*^+/+^, cubilin protein is observed on the apical surface of neuroepithelial cells, while in mutant embryos, cubilin protein is observed predominantly on the basolateral side of the neuroepithelium. Increased expression of cubilin protein is also widely visible throughout the heart and cranial mesenchyme in *RFC1*^-/- ^embryos, and is particularly evident in the region surrounding the notochord. It is of interest to note that staining for cubilin remains high on the apical plasma membrane of the VYS in *RFC1 *mutants, and the polarity of expression does not seem to be altered as it does in the neuroepithelium.

Previous reports suggest that cubilin-mediated endocytosis requires the presence of megalin [47]. If this is the case, even though cubilin is highly expressed in the VYS of *RFC1*^-/- ^embryos, cubilin-mediated internalization of nutrients in the VYS may be impaired in the absence of megalin, resulting in the observed accumulation of cubilin on the apical surface of the plasma membrane. Defective endocytosis of the cubilin-megalin complex and the observed upregulation in gene expression of transferrin, transthyretin, retinol binding protein, and several of the apolipoproteins in *RFC1*^-/- ^embryos may reflect a compensatory upregulation in genes encoding for these cubilin-megalin transported ligands, secondary to a perceived deficiency in these nutrients by the developing embryo.

The expression of *amn *was also significantly upregulated in *RFC1*^-/- ^embryos.*Amn *is expressed in the VE, and forms a tight complex with the N-terminal end of cubilin, providing the transmembrane domain necessary for membrane anchoring and endocytic trafficking of cubilin. *Amn *is therefore an essential component of the cubilin receptor complex, facilitating the endocytosis/transcytosis of cubilin-bound ligands and nutrients that are absorbed from the maternal environment and transported to the developing embryo during gastrulation. Similar to the *RFC1*^-/-^, embryonic development is arrested during gastrulation in *amn *mouse mutants [48-50]. *Amn *knockout embryos appear abnormal during the early primitive streak stage (E6.5–7.0), and then die between E9.5–E10.5. Mutant embryos are smaller than wildtype littermates, and the embryonic ectoderm is underdeveloped [48]. Depending on the genetic background of the *amn *mouse mutant, the amniotic membrane may be present [49] or absent [48]. Extra-embryonic structures derived from proximal streak mesoderm (chorion, allantois, yolk sac blood islands), and distal streak mesoderm (notochord, foregut, cardiac mesoderm) in *amn *mutants appear to develop normally, but embryos have impaired assembly of derivatives of the middle primitive streak, resulting in the absence of paraxial, intermediate and lateral plate mesoderm.

### Dab2

gene expression was upregulated in our microarray analysis of *RFC1*^-/- ^embryos, but the difference was not statistically significant on RT-PCR analysis. *Dab2 *is a cytosolic adapter protein expressed in the VE and VYS. Inactivation of murine *Dab2 *results in disorganization of the VE layer, and early embryonic lethality prior to gastrulation [51]. Embryos appear to develop normally when *Dab2 *is conditionally deleted from only the embryo, indicating that *Dab2 *expression in the VE is the essential component for progression of normal embryogenesis [52]. *Dab2 *binds to a common sequence in the cytoplasmic tails of lipoprotein receptors [53], and has been found in complexes with megalin in the kidney [54,55], suggesting it may be involved in mediating the intracellular trafficking and/or endocytosis of megalin. The *Dab2 *gene is alternatively spliced to produce two protein products [56]; expression of the p96 isoform is essential for normal embryonic development and endocytosis of megalin in the VE [57,58]. In p96 *Dab2 *mutants, megalin and cubilin accumulate at the apical plasma membrane surface, and little intracellular protein or co-localization with early endosome markers is observed. However, if the p67 isoform of *Dab2 *is inactivated, approximately 50% of homozygotes die by E10.5, and surviving mutants are small and developmentally delayed relative to wildtype littermates [58]. Altered expression of *Myo6 *was also detected in our microarray analysis of *RFC1*^-/- ^embryos. Both isoforms of *Dab2 *bind and recruit *Myo6 *to clathrin-coated vesicles [52,59], and function in clathrin-mediated endocytosis in polarized epithelial cells [60-62].

### Receptor-associated protein (RAP or Lrpap1)

is another component of the cubilin-megalin multiligand endocytic receptor complex that demonstrated increased expression in *RFC1*^-/- ^embryos on our microarray analysis, but did not show a statistically significant difference in the RT-PCR analysis. RAP binds both megalin [63] and cubilin [35], and functions as a chaperone-like protein to promote proper folding and protect the ligand binding sites of newly processed low density lipoprotein receptor family proteins, preventing premature interaction of ligands with the receptors [64,65]. Megalin binds to two separate sites on RAP [66] with high affinity, and the binding is calcium-dependent. RAP resides predominantly in the endoplasmic reticulum where it binds and escorts megalin receptors to the golgi. Dissociation of the complex occurs in late endosomes, triggered by the acidic pH of this compartment, and the subsequent protonation of exposed histidine residues on the surface of RAP that modulate the binding/release of RAP from megalin [67]. RAP is subsequently delivered to lysosomes and degraded, while megalin is recycled to the cell surface [68]. RAP deficiency is associated with a decrease in megalin expression and a change in subcellular distribution, causing an accumulation of megalin in intracellular compartments. Homozygous RAP-deficient mice are viable and appear phenotypically normal, although megalin expression in the liver and brain is significantly reduced [69].

In the renal brush border, megalin has been shown to specifically bind NHE3 [70], a Na^+^/H^+ ^ion exchanger that mediates the electroneutral exchange of intracellular H^+ ^for extracellular Na^+ ^across plasma membranes. NHE3 plays a critical role in NaCl homeostasis, and the maintenance of intracellular pH, and has also been shown to play a role in clathrin-mediated endocytosis and endosomal acidification [71]. In renal proximal tubule cells, NHE3 deficiency or inhibition reduces the relative cell surface expression of megalin, suggesting intracellular retention of megalin due to impaired trafficking/recycling of endosomes back to the plasma membrane [71]. On our microarray analysis, the gene expression profile of numerous ion channels was altered in *RFC1*^-/-^embryos (see supplemental data). *RFC1 *functions not only in the cellular uptake of folate, but also as a facilitative anion exchanger, and its deletion may impact intracellular pH homeostasis. The association of RAP with megalin is highly pH dependent. Premature dissociation of the RAP-megalin complex could result in misfolding, or early intracellular degradation of megalin along the secretory pathway, which might provide an alternative explanation for the disappearance of megalin protein on the surface of the VYS and neuroepithelium in *RFC1 *mutants. The absence of cell surface expression of megalin would impact uptake of megalin binding ligands such as transthyretin, hemoglobin, retinol binding protein, apolipoproteins, and shh, and might also impact megalin-dependent endocytosis of the cubilin-binding ligands such as albumin, transferrin, and apolipoprotein A-I. This would result in not only in a folate deficiency, but would also significantly impact maternal-fetal delivery of numerous other nutrients and morphogens required for normal vasculogenesis, hematopoiesis and embryonic development.

## Conclusion

*RFC1 *functions as a facilitative anion exchanger and the primary transporter of reduced folates in multiple tissue types. Polymorphisms in folate transport genes have been implicated as risk factors for certain types of birth defects, and recent evidence from human epidemiological studies demonstrates an association between polymorphisms in *RFC1 *and increased risk for neural tube defects [72-74], and conotruncal heart defects [75,76]. However, the mechanism(s) by which altered *RFC1*-mediated folate transport perturb normal morphogenesis are currently unknown. In our knockout mouse model, loss of functional *RFC1 *during embryogenesis alters the gene expression of numerous transcription factors and ion channels, as well as the expression of a group of genes that code for receptors, ligands and interacting proteins in the cubilin-megalin multiligand endocytic receptor complex. Alterations in the quantity and location of cubilin and megalin protein expression are observed in the yolk sac, neuroepithelium, and mesoderm of E9.5 *RFC1 *nullizygous mutants. Inactivation of *cubilin, amnionless*, or *dab2 *impacts early development, resulting in disorganization of the visceral endoderm, as well as deficiencies in the formation of mesodermal structures, including the chorioallantois, blood islands, and primitive erythrocytes, similar to the phenotype observed in *RFC1*^-/- ^mutants on low dose maternal folate [see Additional file 2]. Inactivation of *lrp2 *(megalin) results in multiple malformations later in embryonic/fetal development that include neuroepithelial defects, and craniofacial and lung dysmorphologies similar to those observed in *RFC1*^-/- ^mutants on high dose maternal folate [see Additional file 2]. The spectrum of malformations observed in *RFC1 *knockout embryos may therefore be causally related to altered expression of the cubilin-amnionless-megalin complex which mediates the maternal-fetal transport of folate and other nutrients, lipids, and morphogens such as sonic hedgehog (Shh) and retinoids that play critical roles in normal embryogenesis.

## Methods

### Experimental animals

*RFC1 *knockout mice were generated by targeted inactivation of the *RFC1 *(*Slc19a1*) allele as previously described [77]. Following identification of germline chimeras, the mutation was transferred from the hybrid stock genetic background (C57BL/6J) onto an inbred SWV genetic background. RFC/SWV heterozygous mice were housed in microisolator cages, maintained on a 12-hour light/dark cycle, and allowed access to normal Harlan-Teklad (Madison, WI) rodent chow [#8604] and autoclaved water in the AAALAC-accredited University of Nebraska Medical Center (UNMC) laboratory animal facility. All animal procedures were done in accordance with the Public Health Service (PHS) policy on Humane Care and Use of Laboratory Animals, and approved by the UNMC Institutional Animal Care and Use committee. Virgin females 50–70 days of age were mated to males between the hours of 5–10 PM each evening. Females exhibiting vaginal plugs at the end of this period were weighed and housed, up to 5 females per cage, and subsequently placed in treatment groups. The time of conception was considered to be 10 PM on the evening of the mating.

### Folate supplementation

Folic acid was purchased from Sigma Chemical Co. (St. Louis, MO), and dissolved in nanopure water to make a solution of the desired concentration. Pregnant dams received folic acid (25 mg/kg) by subcutaneous (S.Q.) injection (0.1 ml/10 gm bwt), beginning on E0.5, and continuing daily throughout gestation until the time of sacrifice.

### Fetal collection and morphological staging

At the desired gestational timepoint (E9.5), pregnant *RFC1*^+/- ^dams were killed by cervical dislocation, the abdomen opened, and the uterine contents removed. The location of all viable fetuses and resorption sites were recorded. Embryos/fetuses were dissected free of the decidual capsule, including its chorion and amnion, and examined for the presence of gross abnormalities. Extraembryonic membranes were collected for genotyping. Whole fetuses were viewed under a Nikon SMZ 1500 stereomicroscope, and photographed using an Optronics^® ^(Goleta, CA) camera.

### Tissue preparation

Three separate litters were collected, and one *RFC1*^+/+ ^and one *RFC1*^-/- ^embryo from each litter were selected for the microarray experiment. Pregnant dams were sacrificed, and E9.5 *RFC1 *embryos were quickly removed from the uterus, placed in RNase-free PBS on ice, weighed, snap frozen and stored at -80°C until RNA extraction was performed. Extraembryonic membranes were collected for genotyping. Following confirmation of genotype, total RNA was extracted from the embryonic tissue, and provided to the UNMC microarray core facility for further processing.

### RNA isolation

Total RNA from each whole E9.5 embryo was isolated using the Arcturus (Mountain View, CA) PicoPure kit with an on-column DNAse treatment following a 30 second homogenization using a Kontes Pellet Pestle. Yields of 1.7–39.6 μg of total RNA were obtained, quantitated, and checked on a NanoChip using a 2100 Bioanalyzer (Agilent Technologies, Palo Alto, CA) in order to determine sample integrity prior to microarray analysis or QRT-PCR. All RNAs were stored at -80°C until downstream analysis was performed. For the microarray experiment, total RNA was reverse-transcribed, and biotin-labeled cRNA probe generated.

### Microarray experiment

100 ng of total RNA was reverse-transcribed and cRNA generated per Affymetrix instructions using the Affymetrix 2-cycle Target Labeling Kit. Resultant cRNA probes were hybridized to the Affymetrix (Santa Clara, CA) Mouse 430 2.0 genome chip, which profiles the expression of 45,101 unique transcripts. Following washing and staining, the chips were scanned using a GeneChip Scanner 3,000 6 G in the UNMC microarray core facility. Images were analyzed using GCOS imaging software. Quality metric parameters including noise level, background, and the efficiency of reverse transcription were ascertained for all hybridizations. Data sets passing the stringent quality recommendations were normalized using the GCOS software, and the raw intensity values exported for further analysis.

### Statistical analysis

#### Microarray data processing

Analyses were conducted with BRB ArrayTools developed by Dr. Richard Simon and Amy Peng. Low-level analysis which converts probe level data to a gene level expression data was done using robust multiarray average (RMA), implemented using the *rma *function of the *Affymetrix *package of the Bioconductor project in the R programming language [78-80]. The RMA background correction method corrects the perfect match (PM) probe intensities by using a model based on the assumption that the observed intensities are the sum of signal and noise. Quantile normalization was used to normalize the PM probes and the calculation of summary expression measures was done using the median polish method, which fits a multichip linear model to the data, and gives the expression on the log_2 _scale. Prior to statistical analysis a filter was applied to reduce the number of genes examined. A gene was excluded unless at least 1 out of the 6 arrays had expression data with a 1.5-fold change or greater in either direction from the gene's median value. This filter reduced the number of transcripts examined to 2982.

#### Differential expression

The mice were paired as litter-mates (one wildtype and one nullizygous embryo from each litter) and for each gene, a random-variance paired t-test was conducted to determine if there was a significant difference in expression between the mutant and wild type groups for that gene [81]. P-value adjustment for multiple comparisons was done with the false discovery rate (FDR) method of Benjamini-Hochberg [82]. A significance level of 0.001 for the adjusted univariate test was used to select differentially expressed genes. This reduced the number of probe sets examined to 288.

### Quantitative real-time PCR (qRT-PCR) validation

#### First-strand cDNA synthesis

The SuperScript™ III First-strand Synthesis System for RT-PCR (Invitrogen, Carlsbad, CA) was used to synthesize first-strand cDNA from total RNA. cDNAs were synthesized starting with 300 ng of sample RNAs or 1000 ng of Mouse Universal RNA (MUR, Stratagene, La Jolla, CA) using oligo(dT) primer according to kit directions. cDNAs were stored at -20°C. Prior to QRTPCR all cDNAs were diluted with nuclease-free water to 10 ng/ul (based on the amount of input RNA).

#### Quantitative RT-PCR

Quantitative RT-PCR was performed using the Stratagene MX3000P with MxPro v 3.00 software for Comparative Quantitation (Stratagene, La Jolla, CA). The 20 μl reaction consisted of 10 μl 2X TaqMan Universal Master Mix with AmpErase UNG (Applied Biosystems, Foster City, CA), 1 μl of 20X Target Mix [mouse gene-specific primers (forward and reverse), 900 nM final concentration; mouse gene-specific 6-FAM-labeled TaqMan MGB probe at 250 nM final concentration] (Applied Biosystems, Foster City, CA), 1 μl of cDNA, 8 μl nuclease-free water (Ambion, Austin, TX). Controls included no-template controls and "Minus reverse transcriptase" controls. All samples were run in triplicate. Two target genes were used for data normalization: Dctn6 (Dynactin 6) and PPIL2 (peptidylprolyl (cyclophilin)-like isomerase 2). See [Additional file 3] for Genes of Interest (GOI) used in the experiments. A calibrator template, Mouse Universal RNA (from mouse tissues, Stratagene, La Jolla, CA) was also used throughout the course of the assays.

#### Statistical analysis of quantitative RT-PCR data

Amplification efficiency curves were generated for each primer/probe set and for each round of cDNA synthesis using 4-fold serial dilutions of mouse universal RNA (Stratagene, La Jolla, CA). Ct values were determined using the MxPro software (Stratagene, La Jolla, CA). Relative levels of mRNA expression in *RFC1 *nullizygous and wildtype embryos were calculated using the Relative Expression Software Tool (REST v1.9.12) [83]. This software provides proper error propagation and robust statistical analysis by using a random reallocation algorithm with 50,000 iterations. Normalization for each experimental sample used the geometric means of the relative concentration of each normalizer gene (Dctn6 and PPIL 2) [84].

### Ingenuity™ Pathway Analysis

The list of significantly altered genes identified in the microarray analysis was also examined for the presence of interacting gene networks using Ingenuity™ Pathway Analysis software. A data set containing gene identifiers and their corresponding expression values was uploaded as an Excel spreadsheet using the template provided in the application. Each gene identifier was mapped to its corresponding gene object in the Ingenuity Pathways Knowledge Base. For our data set, the Affymetrix probe set ID was used as an identifier, and an expression value cutoff of 1.5 was set to identify genes whose expression was significantly differentially regulated. These genes were then used as the starting point for generating biological networks. Biological functions were assigned to each gene network by using the findings that have been extracted from the scientific literature and stored in the Ingenuity Pathways Knowledge Base. The biological functions assigned to each network are ranked according to the significance of that biological function to the network. A Fischer's exact test was used to calculate a p-value determining the probability that the biological function assigned to that network is explained by chance alone.

### Immunohistochemistry

The distribution and abundance of cubilin and megalin were characterized immunohistochemically in placentas and embryos collected from control and folic-acid treated dams. Pregnant females were euthanized on E9.5, embryos collected, fixed in fresh 4% paraformaldehyde, paraffin-embedded, and sectioned at 10 μm. Primary antibodies to cubilin and megalin, and FOLR1 were purchased from Santa Cruz Inc., (Santa Cruz, CA). Primary antibody dilutions were optimized at cubilin (1:100), megalin (1:200), and FOLR1 (1:300). Sections were incubated with biotin-conjugated secondary antibody (light microscopy), followed by incubation with streptavidin-HRP, and diaminobenzidine (DAB) solution until brown staining was visible, and counterstained with methyl green. Sections were viewed under a Nikon Eclipse E800 microscope, photographed using an Optronics^® ^(Goleta, CA) camera and AnalySIS^® ^(Soft Imaging Systems Corp., Lakewood, CA) software, and images processed in Adobe Photoshop.

## List of abbreviations used

***RFC1:****reduced folate carrier 1; ****Shh:****sonic hedgehog; ****Folr1:****folate receptor 1; ****Timd2:****T-cell immunoglobulin and mucin domain-containing 2; ****Myo6:****myosin VI; ****Dab2:****disabled homolog 2; ****Cubn:****cubilin; ****Tf:****transferrin; ****LgalS3: ****lectin, galactoside-binding, soluble, 3; ****Alb:****albumin; ****Gif:****gastric intrinsic factor; ****Amn:****amnionless; ****Lrp2:****megalin; ****Lrpap1:****lipoprotein receptor associated-protein-1 (RAP); ****ScgB1A1:****secretoglobin, family 1A, member 1; ****ApoA1:****apolipoprotein A1; ****ApoE:****apolipoprotein E; ****ApoM:****apolipoprotein M; ****Rbp4:****retinol binding protein 4; ****Ttr:****transthyretin; ****Dctn6:****dynactin 6; ****Ppil2:****peptidylprolyl (cyclophilin)-like isomerase 2; ***VE: **visceral endoderm; **VYS: **visceral yolk sac; **NHE3: **Na+/H+ ion exchanger; **RMA: **robust multiarray average; **FDR: **false discovery rate; **REST: **Relative Expression Software Tool; **DAB: **diaminobenzidine; **HRP: **horseradish peroxidase.

## Authors' contributions

JGvW: experimental design, data interpretation, preparation of figures; writing of manuscript; JM: RNA isolation and preparation of embryo samples for microarray; organization of gene ontology groups (Fig. 1); Ingenuity Pathway analysis (Fig. 2); quantitative RT-PCR validation; LS: statistical analysis of microarray data (Table 1 and supplemental); MvW: statistical analysis of quantitative RT-PCR data (Fig. 4); JW: maintenance of RFC1 mouse colony; timed matings, folate treatments, embryo harvest, graphic design (Fig. 3), photography of immunostained sections (Fig. 5, Fig. 6); JDE: hybridization and scanning of Affymetrix microarray gene chips; LKB: histology, immunohistochemistry; RHF: provided *RFC1 *knockout mouse model; assisted with writing of manuscript. All authors read and approved the final manuscript.

## Supplementary Material

Additional file 1 RFC1 microarray gene table. This table provides a complete list of the 200 known genes that were differentially expressed between E9.5 *RFC1 *nullizygous and wildtype embryos following statistical analysis of the microarray data, including RefSeq transcript ID, gene symbol, description, and fold change for each gene. Click here for file

Additional file 2 Comparison of mouse mutants. This table compares the phenotype of *RFC1 *nullizygous embryos (on low or high dose maternal folate supplementation) with the phenotype of embryos in which different genes in the cubilin-megalin multiligand endocytic receptor complex (ie. *cubilin, amnionless, megalin, Folr1, Dab2, Lrpap1*) have been inactivated. Click here for file

Additional file 3 Quantitative RT-PCR primer/probe list. This table contains a complete list and details regarding the primers/probes used in the qRT-PCR analysis of genes in the multiligand endocytic receptor complex, as well as details on the function of each gene examined. Click here for file
