# Supplementary material for: Microarray analysis of E9.5 reduced folate carrier (RFC1; Slc19a1) knockout embryos reveals altered expression of genes in the cubilin-megalin multiligand endocytic receptor complex
Source: BMC Genomics. 2008 Apr 9;9:156. doi: 10.1186/1471-2164-9-156 (PMC2383917; doi:10.1186/1471-2164-9-156)
Supplement: Additional file 1 — RFC1 microarray gene table. This table provides a complete list of the 200 known genes that were differentially expressed between E9.5 RFC1 nullizygous and wildtype embryos following statistical analysis of the microarray data, including RefSeq transcript ID, gene symbol, description, and fold change for each gene. [file 1471-2164-9-156-S1.doc]

| **Additional File 1: MICROARRAY GENE LIST: *RFC1* nullizygous vs. *RFC1* wildtype** | | | |
| --- | --- | --- | --- |
|  | | | |
| ***RFC, E 9.5, whole embryo, nullizygote vs wildtype, Affymetrix Mouse 430_2 Microarray Results*** | | | |
|  | | | |
| ***RefSeq Transcript ID*** | ***Gene Symbol Affy*** | ***Description*** | ***Fold Change*** |
| ***MULTILIGAND ENDOCYTIC RECEPTOR COMPLEX*** | | | |
| NM_009692 | Apoa1 | apolipoprotein A-I | 65.97 |
| XM_130038 | Cubn | cubilin (intrinsic factor-cobalamin receptor) | 35.49 |
| NM_018816 | Apom | apolipoprotein M | 23.41 |
| NM_013697 | Ttr | transthyretin | 18.02 |
| NM_011255 | Rbp4 | retinol binding protein 4, plasma | 14.43 |
| NM_134249 | Timd2 | T-cell immunoglobulin and mucin domain containing 2 | 12.04 |
| NM_133977 | Trf | transferrin | 9.41 |
| NM_009696 | Apoe | apolipoprotein E | 4.70 |
| NM_001008702 /// NM_023118 | Dab2 | disabled homolog 2 (Drosophila) | 3.78 |
| NM_008662 | Myo6 | myosin VI | 3.56 |
| NM_017399 | Fabp1 | fatty acid binding protein 1, liver | 3.10 |
| NM_013587 | Lrpap1 | low density lipoprotein receptor-related protein associated protein 1 | 2.16 |
| NM_008219 | Hbb-bh1 | hemoglobin Z, beta-like embryonic chain | -25.38 |
| NM_010405 | Hba-x | hemoglobin X, alpha-like embryonic chain in Hba complex | -34.60 |
| NM_008221 | Hbb-y | hemoglobin Y, beta-like embryonic chain | -53.19 |
|  |  |  |  |
| ***TRANSPORT*** | | | |
| ***Lipid Transport*** | | | |
| NM_009692 | Apoa1 | apolipoprotein A-I | 65.97 |
| NM_018816 | Apom | apolipoprotein M | 23.41 |
| NM_009695 | Apoc2 | apolipoprotein C-II | 17.73 |
| NM_023530 | Pla2g12b | phospholipase A2, group XIIB | 9.34 |
| NM_009696 | Apoe | apolipoprotein E | 4.70 |
| NM_008642 | Mttp | microsomal triglyceride transfer protein | 4.36 |
| NM_017399 | Fabp1 | fatty acid binding protein 1, liver | 3.10 |
| NM_013703 | Vldlr | very low density lipoprotein receptor | 2.11 |
| NM_010174 | Fabp3 | fatty acid binding protein 3, muscle and heart | 2.06 |
|  |  |  |  |
| ***Ion Transport/Binding*** | | | |
| ***Calcium*** | | | |
| NM_007595 | Camk2b | Calcium/calmodulin-dependent protein kinase II, beta (Camk2b), mRNA | 2.97 |
| NM_009627 | Adm | adrenomedullin | 2.85 |
| NM_013471 | Anxa4 | annexin A4 | 2.38 |
| NM_009060 | Rgn | regucalcin | 2.27 |
| NM_013472 | Anxa6 | annexin A6 | 1.87 |
| NM_009112 | S100a10 | S100 calcium binding protein A10 (calpactin) | 1.80 |
| NM_010473 | Hrc | histidine rich calcium binding protein | 1.71 |
| NM_019978 | Dcamkl1 | double cortin and calcium/calmodulin-dependent protein kinase-like 1 | -2.31 |
| ***Potassium*** | | | |
| NM_009721 | Atp1b1 | ATPase, Na+/K+ transporting, beta 1 polypeptide | 2.42 |
| XM_355877 | Kcnk6 | potassium inwardly-rectifying channel, subfamily K, member 6 | 1.73 |
| NM_010595 | Kcna1 | Potassium voltage-gated channel, shaker-related subfamily, member 1 (Kcna1), mRNA | -2.21 |
| ***Chloride*** |  |  |  |
| NM_172469 | Clic6 | chloride intracellular channel 6 | 5.60 |
| NM_133648 /// NM_133649 | Slc12a6 | solute carrier family 12, member 6 | 1.82 |
| ***Amino Acid*** | | | |
| NM_011405 | Slc7a7 | solute carrier family 7 (cationic amino acid transporter, y+ system), member 7 | 2.85 |
| ***Carbohydrate*** | | | |
| NM_031197 | Slc2a2 | solute carrier family 2 (facilitated glucose transporter), member 2 | 27.95 |
| XM_283054 | Colec11 | collectin sub-family member 11 | 2.40 |
| NM_011401 | Slc2a3 | solute carrier family 2 (facilitated glucose transporter), member 3 | 2.32 |
|  |  |  |  |
| ***HEMATOPOIESIS/ANGIOGENESIS*** | | | |
| NM_133862 | Fgg | fibrinogen, gamma polypeptide | 15.79 |
| NM_181849 | Fgb | fibrinogen, B beta polypeptide | 9.27 |
| NM_007443 | Ambp | alpha 1 microglobulin/bikunin | 2.93 |
| NM_007709 | Cited1 | Cbp/p300-interacting transactivator with Glu/Asp-rich carboxy-terminal domain 1 | 2.82 |
| NM_133974 | Cdcp1 | CUB domain containing protein 1 | 2.00 |
| NM_178600 | Vkorc1 | vitamin K epoxide reductase complex, subunit 1 | 1.93 |
| NM_013723 | Podxl | podocalyxin-like | 1.89 |
| NM_010612 | Kdr | kinase insert domain protein receptor | 1.79 |
| NM_010848 /// NM_033597 | Myb | myeloblastosis oncogene | -2.17 |
| NM_001025597 /// NM_009578 | Zfpn1a1 | zinc finger protein, subfamily 1A, 1 (Ikaros) | -2.61 |
| NM_020052 | Scube2 | signal peptide, CUB domain, EGF-like 2 | -3.47 |
| NM_007780 | Csf2rb1 | Colony stimulating factor 2 receptor, beta 1, low-affinity (granulocyte-macrophage) (Csf2rb1), mRNA | -3.51 |
| NM_011280 | Trim10 | tripartite motif protein 10 | -4.18 |
| NM_010635 | Klf1 | Kruppel-like factor 1 (erythroid) | -4.57 |
| NM_011465 | Spna1 | spectrin alpha 1 | -5.05 |
| NM_053149 | Hemgn | hemogen | -9.03 |
| NM_011403 | Slc4a1 | solute carrier family 4 (anion exchanger), member 1 | -9.99 |
| NM_133245 | Eraf | erythroid associated factor | -11.27 |
| NM_008219 | Hbb-bh1 | hemoglobin Z, beta-like embryonic chain | -25.38 |
| NM_010405 | Hba-x | hemoglobin X, alpha-like embryonic chain in Hba complex | -34.60 |
| NM_008221 | Hbb-y | hemoglobin Y, beta-like embryonic chain | -53.19 |
|  |  |  |  |
| ***TRANSCRIPTION FACTORS*** | | | |
| ***Homeobox*** | | | |
| NM_011441 | Sox17 | SRY-box containing gene 17 | 2.55 |
| NM_011446 | Sox7 | SRY-box containing gene 7 | 2.36 |
| NM_009234 | Sox11 | SRY-box containing gene 11 | -1.77 |
|  |  |  |  |
| NM_010054 | Dlx2 | distal-less homeobox 2 | -1.75 |
| NM_010053 | Dlx1 | distal-less homeobox 1 | -2.28 |
| NM_008818 | Rhox5 | reproductive homeobox on X chromosome, 5 | 10.50 |
| NM_020496 /// NM_194263 | Tbx20 | T-box 20 | 1.95 |
| NM_008584 | Meox2 | mesenchyme homeobox 2 | -1.95 |
| NM_001025570 /// NM_011127 /// NM_175686 | Prrx1 | paired related homeobox 1 | -2.09 |
| NM_172553 | Cart1 | cartilage homeo protein 1 | -2.12 |
| NM_030708 | Zfhx4 | zinc finger homeodomain 4 | -2.41 |
| NM_010836 | Msx3 | homeo box, msh-like 3 | -2.66 |
| NM_010132 | Emx2 | empty spiracles homolog 2 (Drosophila) | -2.81 |
| NM_001005232 | Dbx1 | developing brain homeobox 1 | -4.01 |
|  |  |  |  |
| ***bHLH*** | | | |
| NM_010419 | Hes5 | hairy and enhancer of split 5 (Drosophila) | -1.97 |
| NM_008506 | Lmyc1 | lung carcinoma myc related oncogene 1 | -1.98 |
| NM_031189 | Myog | myogenin | -2.01 |
| NM_008553 | Ascl1 | achaete-scute complex homolog-like 1 (Drosophila) | -2.03 |
| NM_021560 | Bhlhb5 | basic helix-loop-helix domain containing, class B5 | -2.46 |
| NM_053008 | Olig3 | oligodendrocyte transcription factor 3 | -2.71 |
| NM_009718 | Neurog2 | neurogenin 2 | -3.32 |
| NM_010894 | Neurod1 | neurogenic differentiation 1 | -4.19 |
|  |  |  |  |
| ***Zinc Finger*** | | | |
| NM_009575 | Zic3 | zinc finger protein of the cerebellum 3 | -2.04 |
| NM_027504 | Prdm16 | MKIAA1675 protein | -2.53 |
| NM_001025597 /// NM_009578 | Zfpn1a1 | zinc finger protein, subfamily 1A, 1 (Ikaros) | -2.61 |
| NM_029947 | Prdm8 | PR domain containing 8 | -2.99 |
| NM_009573 | Zic1 | Zinc finger protein of the cerebellum 1 (Zic1), mRNA | -4.06 |
|  |  |  |  |
| ***Other*** | | | |
| NM_016851 /// NM_178083 | Irf6 | interferon regulatory factor 6 | 3.59 |
| NM_008259 | Foxa1 | forkhead box A1 | 2.61 |
| NM_133659 | Erg | Avian erythroblastosis virus E-26 (v-ets) oncogene related (Erg), mRNA | 1.89 |
| NM_011066 | Per2 | period homolog 2 (Drosophila) | 1.77 |
| NM_008901 | Pou3f4 | POU domain, class 3, transcription factor 4 | -1.85 |
| NM_008899 | Pou3f2 | POU domain, class 3, transcription factor 2 | -1.85 |
| NM_010848 /// NM_033597 | Myb | myeloblastosis oncogene | -2.17 |
| NM_145831 | Dmrt2 | doublesex and mab-3 related transcription factor 2 | -2.47 |
| NM_001024918 /// NM_027689 | Rfx4 | regulatory factor X, 4 (influences HLA class II expression) | -2.81 |
| NM_008900 | Pou3f3 | POU domain, class 3, transcription factor 3 (Pou3f3), mRNA | -4.55 |
| NM_010635 | Klf1 | Kruppel-like factor 1 (erythroid) | -4.57 |
|  |  |  |  |
| ***CELL ADHESION*** | | | |
| NM_016675 | Cldn2 | claudin 2 | 8.98 |
| NM_146010 | Tspan8 | tetraspanin 8 | 6.39 |
| NM_018777 | Cldn6 | claudin 6 | 2.74 |
| NM_013505 | Dsc2 | desmocollin 2 | 2.46 |
| NM_007883 | Dsg2 | desmoglein 2 | 2.29 |
| NM_009851 | Cd44 | CD44 antigen | 2.27 |
| NM_010233 | Fn1 | fibronectin 1 | 2.18 |
| NM_008756 | Ocln | occludin | 2.16 |
| NM_026163 | Pkp2 | plakophilin 2 | 1.94 |
| XM_126961 | Plekhh1 | pleckstrin homology domain containing, family H (with MyTH4 domain) member 1 | 1.70 |
| XM_484197 | Itgb8 | PREDICTED: integrin beta 8 [Mus musculus], mRNA sequence | -1.73 |
| NM_007495 | Astn1 | astrotactin 1 | -2.15 |
| NM_023844 | Jam2 | junction adhesion molecule 2 | -2.55 |
|  |  |  |  |
| ***CYTOSKELETON*** | | | |
| NM_008471 | Krt1-19 | keratin complex 1, acidic, gene 19 | 2.54 |
| NM_010664 | Krt1-18 | keratin complex 1, acidic, gene 18 | 2.18 |
| NM_031170 | Krt2-8 | keratin complex 2, basic, gene 8 | 2.13 |
| NM_009510 | Vil2 | villin 2 | 2.04 |
| NM_011526 | Tagln | transgelin | 1.99 |
| NM_007599 | Capg | capping protein (actin filament), gelsolin-like | 1.88 |
| NM_010859 | Myl3 | myosin, light polypeptide 3 | 1.84 |
| --- | Mtap2 | microtubule-associated protein 2 | -1.99 |
| NM_023279 | Tubb3 | tubulin, beta 3 | -2.97 |
| NM_008691 | Nef3 | neurofilament 3, medium | -3.39 |
|  |  |  |  |
| ***PROTEOLYSIS*** | | | |
| NM_007801 | Ctsh | cathepsin H | 10.41 |
| NM_015775 | Tmprss2 | transmembrane protease, serine 2 | 8.54 |
| NM_010074 | Dpp4 | dipeptidylpeptidase 4 | 7.40 |
| NM_023270 | Rnf128 | ring finger protein 128 | 3.41 |
| XM_125830 | Cpm | carboxypeptidase M | 2.66 |
| NM_022325 | Ctsz | cathepsin Z | 2.36 |
| NM_009244 | Serpina1b | serine (or cysteine) preptidase inhibitor, clade A, member 1b | 2.34 |
| NM_133351 | Prss8 | protease, serine, 8 (prostasin) | 2.30 |
|  |  |  |  |
| ***APOPTOSIS/CELL CYCLE ARREST*** | | | |
| NM_028608 | Glipr1 | GLI pathogenesis-related 1 (glioma) | 3.15 |
| NM_139198 | Plac8 | placenta-specific 8 | 2.60 |
| NM_007669 | Cdkn1a | cyclin-dependent kinase inhibitor 1A (P21) | 2.40 |
| NM_019521 | Gas6 | growth arrest specific 6 | 1.99 |
| NM_007831 | Dcc | Deleted in colorectal carcinoma (Dcc), mRNA | -2.74 |
|  |  |  |  |
| ***SIGNAL TRANSDUCTION*** | | | |
| XM_130346 | Gpr155 | G protein-coupled receptor 155 | 4.73 |
| NM_007616 | Cav1 | caveolin, caveolae protein 1 | 2.55 |
| NM_019840 | Pde4b | phosphodiesterase 4B, cAMP specific | 2.49 |
| XM_132099 | Centd1 | centaurin, delta 1 | 2.45 |
| NM_018750 | Rassf5 | Ras association (RalGDS/AF-6) domain family 5 | 2.34 |
| NM_011212 | Ptpre | protein tyrosine phosphatase, receptor type, E | 2.23 |
| NM_029352 | Dusp9 | dual specificity phosphatase 9 | 2.04 |
| NM_017469 | Gucy1b3 | guanylate cyclase 1, soluble, beta 3 | 1.97 |
| NM_133222 | Eltd1 | EGF, latrophilin seven transmembrane domain containing 1 | 1.94 |
| NM_001005784 /// NM_001005787 /// NM_007704 /// NM_172696 | Inadl | InaD-like (Drosophila) | 1.80 |
| NM_177355 | Plcxd3 | phosphatidylinositol-specific phospholipase C, X domain containing 3 | -1.82 |
| NM_008987 | Ptx3 | pentraxin related gene | -2.28 |
| NM_172815 | Rspo2 | R-spondin 2 homolog (Xenopus laevis) | -2.35 |
| NM_138683 | Rspo1 | R-spondin homolog (Xenopus laevis) | -2.48 |
| NM_019985 | Clec1b | C-type lectin domain family 1, member b | -2.53 |
| NM_025285 | Stmn2 | stathmin-like 2 | -2.93 |
| NM_009133 | Stmn3 | stathmin-like 3 | -3.35 |
|  |  |  |  |
| ***GROWTH FACTORS*** | | | |
| NM_008343 | Igfbp3 | insulin-like growth factor binding protein 3 | 2.05 |
| NM_016719 | Grb14 | growth factor receptor bound protein 14 | 1.93 |
| NM_010515 | Igf2r | Insulin-like growth factor 2 receptor (Igf2r), mRNA | 1.74 |
|  |  |  |  |
| ***NEURONAL DEVELOPMENT*** | | | |
| XM_138955 | Sema3g | sema domain, immunoglobulin domain (Ig), short basic domain, secreted, (semaphorin) 3G | 1.88 |
| NM_008629 | Msi1h | Musashi homolog 1(Drosophila) (Msi1h), mRNA | -1.72 |
| NM_001009929 /// NM_001009930 /// NM_029426 | Brsk2 | BR serine/threonine kinase 2 | -1.94 |
| NM_008973 | Ptn | pleiotrophin | -2.08 |
| NM_175499 | Slitrk6 | SLIT and NTRK-like family, member 6 | -2.10 |
| NM_080448 /// NM_153070 | Srgap3 | SLIT-ROBO Rho GTPase activating protein 3 | -2.41 |
| NM_011993 | Dpysl4 | dihydropyrimidinase-like 4 | -2.64 |
| NM_183171 | Fez1 | fasciculation and elongation protein zeta 1 (zygin I) | -2.87 |
| NM_010488 | Elavl4 | ELAV (embryonic lethal, abnormal vision, Drosophila)-like 4 (Hu antigen D) | -3.51 |
| NM_010750 | Mab21l1 | mab-21-like 1 (C. elegans) | -2.41 |
|  |  |  |  |
| ***IMMUNE FUNCTION*** | | | |
| NM_010016 | Daf1 | decay accelerating factor 1 | 2.30 |
| NM_025378 | Ifitm3 | interferon induced transmembrane protein 3 | 2.17 |
|  |  |  |  |
| ***RNA PROCESSING*** | | | |
| NM_130888 | Nxf7 | nuclear RNA export factor 7 | 5.85 |
| NM_021472 /// NM_201239 | Rnase4 | ribonuclease, RNase A family 4 | 3.73 |
| NM_009477 | Upp1 | uridine phosphorylase 1 | 2.31 |
| XM_356586 | Nova1 | neuro-oncological ventral antigen 1 | -2.05 |
| XM_484289 | Ell2 | elongation factor RNA polymerase II 2 | -2.24 |
|  |  |  |  |
| ***ONE-CARBON METABOLISM*** | | | |
| NM_007607 | Car4 | carbonic anhydrase 4 | 3.84 |
| NM_022884 | Bhmt2 | betaine-homocysteine methyltransferase 2 | 2.75 |
|  |  |  |  |
| ***OTHER*** | | | |
| NM_011635 | Trap1a | tumor rejection antigen P1A | 24.92 |
| NM_007423 | Afp | alpha fetoprotein | 20.69 |
| NM_029269 | Spp2 | secreted phosphoprotein 2 | 17.28 |
| NM_009258 | Spink3 | serine peptidase inhibitor, Kazal type 3 | 13.54 |
| NM_021480 | Tdh | L-threonine dehydrogenase | 7.40 |
| NM_009434 | Phlda2 | pleckstrin homology-like domain, family A, member 2 | 6.05 |
| NM_175540 | Eda2r | ectodysplasin A2 isoform receptor | 3.43 |
| NM_029413 | Morc4 | microrchidia 4 | 3.00 |
| NM_145419 | Hkdc1 | hexokinase domain containing 1 | 2.50 |
| NM_029508 | Pcgf5 | polycomb group ring finger 5 | 2.42 |
| NM_013560 | Hspb1 | heat shock protein 1 | 2.32 |
| NM_009546 | Trim25 | tripartite motif protein 25 | 2.31 |
| NM_001005863 /// NM_001005864 /// NM_001005865 /// NM_178902 | Mtus1 | mitochondrial tumor suppressor 1 | 2.30 |
| NM_177025 | Cobll1 | Cobl-like 1 | 2.25 |
| NM_007646 | Cd38 | CD38 antigen | 2.20 |
| NM_023663 | Ripk4 | receptor-interacting serine-threonine kinase 4 | 2.13 |
| NM_011656 | Tuft1 | tuftelin 1 | 1.85 |
| NM_144513 | Gtl2 | GTL2, imprinted maternally expressed untranslated mRNA | 1.82 |
| NM_010893 | Neu1 | Neuramidase 1 | 1.82 |
| NM_023122 | Gpm6b | glycoprotein m6b | -1.71 |
| NM_009130 | Scg3 | secretogranin III | -1.80 |
| XM_128530 | Fbxl16 | F-box and leucine-rich repeat protein 16 | -1.99 |
| NM_008516 | Lrrn1 | leucine rich repeat protein 1, neuronal | -3.52 |
| NM_009221 | Snca | synuclein, alpha | -4.84 |
| NM_007986 | Fap | fibroblast activation protein | -5.12 |
