# Supplementary material for: Microarray analysis of E9.5 reduced folate carrier (RFC1; Slc19a1) knockout embryos reveals altered expression of genes in the cubilin-megalin multiligand endocytic receptor complex
Source: BMC Genomics. 2008 Apr 9;9:156. doi: 10.1186/1471-2164-9-156 (PMC2383917; doi:10.1186/1471-2164-9-156)
Supplement: Additional file 2 — Comparison of mouse mutants. This table compares the phenotype of RFC1 nullizygous embryos (on low or high dose maternal folate supplementation) with the phenotype of embryos in which different genes in the cubilin-megalin multiligand endocytic receptor complex (ie. cubilin, amnionless, megalin, Folr1, Dab2, Lrpap1) have been inactivated. [file 1471-2164-9-156-S2.doc]

**Additional File 2: COMPARISON OF MOUSE MUTANTS**

| **Mutant** | **Cubilin** | **Amnionless** | **Dab2** | **RFC1**  (low folate) | **RFC1**  (high folate) | **Folr1** | **Lrp2 (Megalin)** | **Lrpap1 (RAP)** |
| --- | --- | --- | --- | --- | --- | --- | --- | --- |
|  |  |  |  |  |  |  |  |  |
| Embryonic Survival | E7.5-13.5^1^  Developmental arrest at E8-8.5 | E8.5-10.5^2-4^ | E4.5-6.5^5^  E5.5-9.5^6^ | E4.5-6.5  (no folate)  E9.5-10.5  (low folate) |  | E10.5  (no folate)^9^ |  |  |
| Visceral Endoderm (VE) | Disorganized VE; loss of apical vesicles |  | Disorganized VE; dispersed; loss of polarity |  |  |  |  |  |
| Amnion |  | Presence depends on genetic background | ^5^Amniotic cavitation not initiated  ^6^Amnion absent | Amnion present |  |  |  |  |
| Chorion | Defective chorioallantoic fusion |  | ^6^Chorion and allantois absent | Defective chorioallantoic fusion |  |  |  |  |
| Yolk Sac Blood Islands; Primitive Erythropoiesis | Large blood islands, vessels fail to undergo remodeling; abnormal RBC morphology |  |  | Blood islands form;  failure of primitive erythropoiesis; megalin expression absent in VYS |  |  |  |  |
| Mesoderm | Deficiencies in paraxial mesoderm; no somites | Deficiencies in paraxial and lateral plate mesoderm; no somites |  | Deficiencies in mesoderm formation | Deficiencies in mesodermal structures |  |  |  |
| Neural Tube |  |  |  | Thin neuroepithelium; craniorachischisis | Thin neuroepithelium; Exencephaly | Exencephaly; cranio-  Rachischisis^9,10^ | Thin neuroepithelium; Holoprosen-cephaly |  |
| Organogenesis |  |  |  |  | Craniofacial, heart, lung, eye, skin malformations | Craniofacial, eye, heart malformations^11^ | Craniofacial, eye, lung, kidney malformations |  |
| Postnatal Viability |  |  |  |  | E18.5  (high folate)  PND 1-12  (high folate) | E18.5  (low folate)  Viable adults  (high folate) | Die within 2-3 hours perinatally from respiratory failure^7^ | Viable; phenotypically normal adults;  ↓ Lrp2 expression in liver and brain^8^ |

**REFERENCES**

**^1^**Smith, B.T., Mussell, J.C., Fleming, P.A., Barth, J.L., Spyropoulos, D.D., Cooley, M.A., Drake, C.J., and Argraves, W.S. 2006. Targeted disruption of cubilin reveals essential developmental roles in the structure and function of endoderm and in somite formation. *BMC Dev Biol* **6**: 30.

**^2^**Wang, X., Bornslaeger, E.A., Haub, O., Tomihara-Newberger, C., Lonberg, N., Dinulos, M.B., Disteche, C.M., Copeland, N., Gilbert, D.J., Jenkins, N.A., and Lacy, E. 1996. A candidate gene for the amnionless gastrulation stage mouse mutation encodes a TRAF-related protein. *Dev Biol* **177**(1): 274-290.

**^3^**Tomihara-Newberger, C., Haub, O., Lee, H.G., Soares, V., Manova, K., and Lacy, E. 1998. The amn gene product is required in extraembryonic tissues for the generation of middle primitive streak derivatives. *Dev Biol* **204**(1): 34-54.

^4^Kalantry, S., Manning, S., Haub, O., Tomihara-Newberger, C., Lee, H.G., Fangman, J., Disteche, C.M., Manova, K., and Lacy, E. 2001. The amnionless gene, essential for mouse gastrulation, encodes a visceral-endoderm-specific protein with an extracellular cysteine-rich domain. *Nat Genet* **27**(4): 412-416.

^5^Yang, D.H., Smith, E.R., Roland, I.H., Sheng, Z., He, J., Martin, W.D., Hamilton, T.C., Lambeth, J.D., and Xu, X.X. 2002. Disabled-2 is essential for endodermal cell positioning and structure formation during mouse embryogenesis. *Dev Biol* **251**(1): 27-44.

^6^Morris, S.M., Arden, S.D., Roberts, R.C., Kendrick-Jones, J., Cooper, J.A., Luzio, J.P., and Buss, F. 2002. Myosin VI binds to and localises with Dab2, potentially linking receptor-mediated endocytosis and the actin cytoskeleton. *Traffic* **3**(5): 331-341.

^7^Willnow, T.E., Hilpert, J., Armstrong, S.A., Rohlmann, A., Hammer, R.E., Burns, D.K., and Herz, J. 1996. Defective forebrain development in mice lacking gp330/megalin. *Proc Natl Acad Sci U S A* **93**(16): 8460-8464.

^8^Willnow, T.E., Armstrong, S.A., Hammer, R.E., and Herz, J. 1995. Functional expression of low density lipoprotein receptor-related protein is controlled by receptor-associated protein in vivo. *Proc Natl Acad Sci U S A* **92**(10): 4537-4541.

^9^Piedrahita, J.A., Oetama, B., Bennett, G.D., van Waes, J., Kamen, B.A., Richardson, J., Lacey, S.W., Anderson, R.G., and Finnell, R.H. 1999. Mice lacking the folic acid-binding protein Folbp1 are defective in early embryonic development. *Nat Genet* **23**(2): 228-232.

^10^Tang LS, and Finnell, R.H. 2003. Neural and orofacial defects in Folp1 knockout mice [corrected]. *Birth Defects Res A Clin Mol Teratol* **67**(4): 209-218.

^11^Tang, L.S., Wlodarczyk, B.J., Santillano, D.R., Miranda, R.C., and Finnell, R.H. 2004. Developmental consequences of abnormal folate transport during murine heart morphogenesis. *Birth Defects Res Part A Clin Mol Teratol* **70**(7): 449-458.
