# Supplementary material for: Microarray analysis of E9.5 reduced folate carrier (RFC1; Slc19a1) knockout embryos reveals altered expression of genes in the cubilin-megalin multiligand endocytic receptor complex
Source: BMC Genomics. 2008 Apr 9;9:156. doi: 10.1186/1471-2164-9-156 (PMC2383917; doi:10.1186/1471-2164-9-156)
Supplement: Additional file 3 — Quantitative RT-PCR primer/probe list. This table contains a complete list and details regarding the primers/probes used in the qRT-PCR analysis of genes in the multiligand endocytic receptor complex, as well as details on the function of each gene examined. [file 1471-2164-9-156-S3.doc]

**Additional File 3: ENDOCYTIC RECEPTOR COMPLEX qRT-PCR PRIMER/PROBE LIST**

|  | *RFC1* qRT-PCR primers/probes |  | |  | |
| --- | --- | --- | --- | --- | --- |
| *Gene Symbol* | *Gene* | *Applied Biosystems TaqMan® Gene Expression Assays* | | | *Description/ Function* |
| Amn | Amnionless | Mm00473870_m1 | GOI | | integral membrane protein; forms a tight complex with the N-terminal end of cubilin and provides the transmembrane domain necessary for membrane anchoring and endocytic trafficking of cubilin; role in embryogenesis |
| ApoA1 | Apolipoprotein A-I | Mm00437569_m1 | GOI | | lipid metabolism and transport; binds cubilin with high affinity; internalized via endocytosis of cubilin-amnionless complex |
| Cubn | Cubilin | Mm01325078_m1 | GOI | | peripheral membrane protein; forms a complex with amnionless; binds intrinsic factor-vitamin B12 and transferrin; binds Lrpap1 (RAP) and interacts with megalin receptor in mediating multiligand endocytosis; role in embryogenesis |
| Dab2 | Disabled homolog 2 | Mm00517751_m1 | GOI | | cytosolic adaptor protein that binds the cytoplasmic tail of megalin; interacts with Myo6 in mediating endocytosis of clathrin-coated pits; role in early embryogenesis |
| Folr1 | Folate receptor 1 | Mm00433355_m1 | GOI | | high affinity folate receptor; GPI-anchored and soluble form; megalin binds the soluble form of Folr1; role in embryogenesis |
| Lrp2 | Low density lipoprotein receptor-related protein 2 (Lrp2; Megalin) | Mm01328172_g1 | GOI | | multiligand endocytic receptor; mediates uptake of soluble Folr1, retinol binding protein, transthyretin, sonic hedgehog, and apolipoproteins E and M, among others; important for mediating endocytosis of cubilin; role in embryogenesis |
| Lrpap1 | Low density lipoprotein receptor-related protein associated protein 1 (RAP) | Mm00660272_m1 | GOI | | binds megalin and cubilin; acts as chaperone to promote proper folding and stability of megalin; protects megalin from early binding of ligands that would result in ER retention and impaired intracellular trafficking; RAP-megalin association is pH dependent |
| Rbp4 | Retinol Binding Protein 4 | Mm00803266_m1 | GOI | | retinoid binding and transport; ligand for megalin |
| Shh | Sonic hedgehog | Mm00436527_m1 | GOI | | Important morphogen in embryogenesis; cell signaling; important for specification of ventral neural tube and forebrain progenitor domains; megalin functions as an endocytic receptor for Shh |
| Timd2 | T-cell immunoglobulin and mucin domain containing 2 | Mm00506693_m1 | GOI | | receptor for H-ferritin endocytosis; H-ferritin subunit levels are regulated by vitamin B12 availability |
| Trf | Transferrin | Mm00446708_m1 | GOI | | iron transport and homeostasis; cubilin ligand |
| Ttr | Transthyretin | Mm00443267_m1 | GOI | | hormone generation and transport, retinoid binding, ligand for megalin |
| Dctn6 | Dynactin 6 | Mm00495994_m1 | HKG | | mitochondrion organization and biogenesis, lipid biosynthesis, cytoskeleton |
| Ppil2 | Peptidylprolyl isomerase (cyclophilin)-like 2 | Mm00713185_m1 | HKG | | protein folding and ubiquitination |
